# Rapid monocyte infiltration following retinal detachment is dependent on non-canonical IL6 signaling through gp130

**DOI:** 10.1186/s12974-017-0886-6

**Published:** 2017-06-23

**Authors:** Xinlei Wang, Eric B. Miller, Mayank Goswami, Pengfei Zhang, Kaitryn E. Ronning, Sarah J. Karlen, Robert J. Zawadzki, Edward N. Pugh, Marie E. Burns

**Affiliations:** 1 Department of Ophthalmology & Vision Science, University of California, Davis, USA; 2 UC Davis RISE Eye-Pod Laboratory, University of California, Davis, USA; 3 Center for Neuroscience, 1544 Newton Court, Davis, CA 95618 USA; 4 Department of Cell Biology and Human Anatomy, University of California, Davis, USA

**Keywords:** Retinal detachment, Monocyte, Interleukin 6, IL6 Rα, gp130, CCL2, Microglia, Retinal degeneration, Inflammation

## Abstract

**Background:**

Retinal detachment (RD) can lead to proliferative vitreoretinopathy (PVR), a leading cause of intractable vision loss. PVR is associated with a cytokine storm involving common proinflammatory molecules like IL6, but little is known about the source and downstream signaling of IL6 and the consequences for the retina. Here, we investigated the early immune response and resultant cytokine signaling following RD in mice.

**Methods:**

RD was induced in C57BL/6 J and IL6 knockout mice, and the resulting inflammatory response was examined using immunohistochemistry and flow cytometry. Cytokines and signaling proteins of vitreous and retinas were quantified by multiple cytokine arrays and Western blotting. To attempt to block IL6 signaling, a neutralizing antibody of IL6 receptor α (IL6Rα) or IL6 receptor β (gp-130) was injected intravitreally immediately after RD.

**Results:**

Within one day of RD, bone marrow-derived Cd11b + monocytes had extravasated from the vasculature and lined the vitreal surface of the retina, while the microglia, the resident macrophages of the retina, were relatively unperturbed. Cytokine arrays and Western blot analysis revealed that this sterile inflammation did not cause activation of IL6 signaling in the neurosensory retina, but rather only in the vitreous and aqueous humor. Monocyte infiltration was inhibited by blocking gp130, but not by IL6 knockout or IL6Rα blockade.

**Conclusions:**

Together, our results demonstrate that monocytes are the primary immune cell mediating the cytokine storm following RD, and that any resulting retinal damage is unlikely to be a direct result of retinal IL6 signaling, but rather gp130-mediated signaling in the monocytes themselves. These results suggest that RD should be treated immediately, and that gp130-directed therapies may prevent PVR and promote retinal healing.

**Electronic supplementary material:**

The online version of this article (doi:10.1186/s12974-017-0886-6) contains supplementary material, which is available to authorized users.

## Background

Retinal detachment (RD) refers to separation of the neurosensory retina from the underlying retinal pigment epithelium (RPE) in the posterior eye. RD occurs spontaneously with a prevalence of 1–2.2 in 10,000 [1–4], and much more frequently in elderly patients after cataract surgery (0.99%) and in high myopia populations [5]. Prompt surgical procedures are required to save vision, but are often delayed for the convenience of both patient and practitioner. In many countries, this delay exceeds several weeks [6]. Delayed treatment often leads to serious secondary complications, including proliferative vitreoretinopathy (PVR). PVR is caused by proliferation of glial or RPE cells to form a fibrous, multicellular scar termed epiretinal membrane at the retinal surface. The epiretinal membrane can produce physical traction and deformation of the retina, leading to more widespread detachment, tears, and retinal puckers. Because PVR often leads to poor visual outcomes, understanding how PVR develops and developing preventative approaches is of utmost importance.

PVR is triggered by a cytokine storm, including MCP-1/CCL2 [7], IL6 [7–9], ICAM-1 [8], and TIMP-1 [9]. One common pro-inflammatory cytokine implicated in PVR, as well as the acute phase response of many inflammatory diseases, is interleukin 6 (IL6). Secreted by T cells, dendritic cells and macrophages, IL6 is significantly elevated in the vitreous [9, 10], aqueous humor [7] and subretinal fluid [7–9] of PVR patients in a manner correlated with the extent of the RD [9]. IL6 receptors consist of subunit α (IL6Rα) and subunit β (gp130, also known as IL6Rβ and CD130), a signal transducer also shared by many other cytokines. IL6Rα is a plasma membrane protein expressed by hepatocytes and some leukocytes, though a soluble form of IL6Rα (sIL6Rα) comprising the extracellular portion of the receptor can bind IL6 with a similar affinity. Both humanized IL6-specific mAb and IL6Rα-specific mAb, which target IL6 and sIL6Rα respectively, have been shown to be effective in treating rheumatoid arthritis and experimental colitis [11].

In the present study, we investigate the acute inflammation triggered by RD, and test the role of IL6 signaling in its initiation and resolution. These findings have important implications for treatment of RD in humans, and may motivate future scientific research on cytokine-specific antibody development for preventing PVR.

## Methods

### Animals and study design

All animals were handled in accordance with NIH guidelines for the care and use of experimental animals and approved protocol by the Institutional Animal Care and Use Committee of the University of California, Davis. Adult (8-week-old) C57BL/6 J mice and IL6 knockout (KO) mice were purchased from Jackson Laboratory (stocks 000664, 002650). For ocular injections and *in vivo* imaging, mice were anesthetized with 1-4% isoflurane, and the pupils dilated with 1% tropicamide and 2.5% phenylephrine ophthalmic solution. Lubricant eye gel (GenTeal) was used to maintain corneal hydration during the procedures. For tissue collection, mice were euthanized by CO_2_ narcosis.

Mice were randomized into 5 groups for analysis: (1) Normal control with no ocular perturbations (2) RD in both eyes with no treatment; (3) RD with intravitreal injection of anti-IL6Rα antibody in both eyes; (3) RD with intravitreal injection of anti-gp130 antibody in both eyes and (4) RD with intravitreal injection of IgG1 isotype control or PBS in both eyes; (5) IL6 KO mice were induced RD without any treatment or intravitreal injection.

### Subretinal and intravitreal Injection

A previous method [12] for inducing bullous and permanent RD was modified to avoid any retinal holes and subretinal hemorrhage. Briefly, a superior scleral hole was gently made with the bevel of 30 G insulin syringe needle (BD Ultra-Fine™), avoiding any retinal damage. Anterior chamber puncture was made to relieve any elevation of intraocular pressure. A 33 G needle connected to Hamilton 2.5 μl syringe was inserted into the scleral hole and carefully positioned within the subretinal space. Sodium hyaluronate (2 μl, ProVisc, Alcon) was gently injected to detach the neurosensory retina from RPE. In the treated groups, 2 μl of polyclonal goat anti-mouse IL6Rα IgG antibody (R&D system, AF1830) or monoclonal rat anti-mouse gp130 IgG1 antibody (R&D system, MAB4682) or monoclonal mouse IgG1 isotype control (R&D system, MAB002) or PBS was injected into the vitreous cavity following detachment. The success of hemi-retinal detachment was confirmed by visual microscopic inspection, and in a subset of cases, OCT imaging. Mice with detachments that were accompanied by retinal holes, subretinal or vitreous hemorrhage were excluded from subsequent studies. More details about anti-IL6Rα and anti-gp130antibodies used in the current study are given in Additional file 1: Table S1.

### OCT imaging

OCT imaging was performed using a custom imaging system as previously described [13]. OCT volumes consisting of 100 B-scans (2000 A-scans/B-scan) spanning ∼ 1.6 mm × 1.6 mm at the retina (32 μm/deg) were used to quantify changes associated with retinal detachment. Six consecutive B-scans were averaged to reduce the speckle noise in the image, corresponding to lateral averaging over 80 μm. Retinal thickness was extracted using semi-automated segmentation software utilizing support vector machine [14].

### Aqueous humor, vitreous and retina collection

After euthanasia, eyes were removed from the orbit. A limbus incision was made with the bevel of 30G needle, then the tip of a 2.5 μl pipette was inserted into the vitreous cavity to collect aqueous and vitreous humor (typically, 3–5 μl from each eye). Anterior segments were removed and discarded, and retinas were separated from the RPE. Retina and vitreous samples were snap frozen in liquid nitrogen and stored in −80 °C for further use.

### Immunohistochemistry

Eyes were fixed in 4% paraformaldehyde in PBS for 5 min at room temperature. The cornea and lens were then removed with the vitreous still attached. The eyecups were fixed for another 20 min, and then dehydrated with 30% sucrose at 4 °C overnight. Eyecups were embedded in OCT compound (Tissue-Tek, Sakura) at −20 °C, then sectioned through the optic nerve at a thickness of 20 μm in the sagittal plane using Microm HM 550 cryostat (Thermo Scientific). For retinal flat mounts, nasal-temporal cuts were made along the two large vessels at the posterior surface for orientation and relaxing cuts were made to divide the inferior retina into three quadrants. Tissue was incubated with 1% Triton X-100 for 30 min at room temperature, and blocked with 1.5% BSA (bovine serum albumin, A7030, Sigma-Aldrich) at 37 °C for 60 min. Tissue was then incubated with rabbit anti-Iba1 (1:100, Wako) and rat anti-mouse CD11b (1:100, eBioscience) overnight at 4 °C. Secondary staining with Alexa 488-conjugated goat anti-rabbit and Alexa 633-conjugated goat anti-rat antibodies (both 1:300, Invitrogen Life Technologies) was performed at 37 °C for 60 min. Tissue was mounted with ProLong gold antifade reagent (Invitrogen Life Technologies) and imaged using a Nikon Ti-E A1 multiphoton imaging system. More details about the anti-IL6Rα antibodies and anti-gp130 antibody used for supplementary immunohistochemistry experiments were shown in Additional file 1: Table S1.

### Flow cytometry

Cells were harvested and counted as described previously [15] with some modifications. Briefly, two retinas were minced in 1 ml of Hank’s Balanced Salt Solution (Thermo Fisher Scientific) containing 2 mg/ml Collagenase D and 28 U/ml DNase I (both from Sigma-Aldrich). Tissue was incubated at 37 °C for 60 min, and re-suspended in 2 ml of Hibernate media (Thermo Fisher Scientific). The suspension was filtered through a 140 μm-mesh screen (Sigma-Aldrich), washed, and centrifuged; supernatant was carefully removed. Cell pellets were fixed and permeabilized for 10 min at RT in 4% paraformaldehyde/0.1% saponin buffer (Sigma-Aldrich), then blocked for 15 min at RT in 3% BSA. Cells were incubated in Alexa Fluor 647 conjugated anti-mouse CD11b antibody (1:100, Biolegend, USA) and Alexa Fluor 488 conjugated IB4 (1:100, Thermo Fisher Scientific) overnight at 4 °C. Suspensions were finally washed and re-suspended in 300 μl PBS. Data were acquired on a BD FACScan flow cytometer (BD Biosciences), 10,000 events were collected for each sample and then analyzed using the FlowJo software (Tree Star).

### Multiple cytokine arrays

Retinas were homogenized in PBS with protease inhibitors (cOmplete, mini, EDTA-free cocktail tablets, Roche) and centrifuged at 10,000 g for 5 min to remove cellular debris. Protein concentrations were determined using total protein assay (Pierce 660 nm Protein Assay Kit, Thermo Fisher Scientific) and then retinal lysates (150 μg) were incubated with cytokine array membranes (Panel A, R&D System) according to the manufacturer’s protocol. Membranes were developed with 800CW streptavidin (IRDye, LI-COR) for 30 min, and imaged using a LI-COR Odyssey system. Images were analyzed with Image Studio Lite 4.0 software (LI-COR). Fluorescence intensities for each cytokine were averaged and normalized relative to healthy normal retinas.

### Western blotting

To prepare whole retinal lysates, 3 retinas from different mice were homogenized in 100 μl lysis buffer (9803S, Cell Signaling Technology) and centrifuged; clear supernatants were isolated, and protein concentrations were determined using total protein assay. Aqueous humor/vitreous samples from 10 eyes were mixed and diluted in lysis buffer (1:10) with gentle tricheration. Equivalent proteins of retinal homogenates (120 μg) or aqueous humor/vitreous mix (60 μg) were separated by electrophoresis (Mini-Protean TGX precast protein gels, BIO-RAD), and transferred onto PVDF membrane (Immun-Blot, BIO-RAD). After blocking (Odyssey TBS Blocking Buffer, LI-COR), the membranes were incubated overnight at 4 °C with primary antibody: rat anti-IL6Rα monoclonal antibody (1:100, extracellular domain, LS-C70920, LifeSpan BioSciences); rabbit anti-gp130 polyclonal antibody (1:100, M-20, SC-656, Santa Cruz); rabbit anti-beta actin antibody (1:1000, ab8227, Abcam); rabbit anti-phospho-STAT3 (Tyr705) antibody (1:500, #9131, Cell Signaling Technology); rabbit anti-SHP2 antibody (1:100, C-18, sc-280, Santa Cruz); goat anti-SOCS3 antibody (1:100, M-20, sc-7009, Santa Cruz); or PathScan® PDGFR activity assay (1:100, phospho-PDGFR, phospho-SHP2, phospho-Akt, and phospho-p44/42 Erk1/2 Multiplex Western Detection Cocktail II, #5304, Cell Signaling Technology). Membranes were washed, then incubated with 680LT goat anti-rabbit and 800CW goat anti-rat secondary antibodies (both 1:10,000, IRDye, LI-COR) for 30 min at RT. Immunoreactive bands were visualized and analyzed by Odyssey Imaging System and Image Studio Lite 4.0 software (LI-COR). More details about anti-IL6Rα and anti-gp130 antibodies used in the current study were shown in Additional file 1: Table S1.

### Statistical analysis

Throughout, data are presented as mean ± standard error, unless otherwise specified. Multiple-group comparisons of flow cytometry data were performed using two-way ANOVA followed by Tukey Honest Significant Differences test, and two-group comparisons were performed using two-sided unpaired Welch Two Sample t-tests. When data were highly positively skewed, the distributions were first logarithmically transformed. Statistics were run on the results before and after logarithmic transformation, and when minor differences were observed the results of the transformed data were used. Transformed data were never directly compared to non-transformed data. Statistical analyses of flow cytometry data were performed using R version 3.3.1 (R Core Team 2016). The normalized folds in results of multiple cytokine arrays and Western blots were analyzed using one-way ANOVA followed by Tukey-Kramer adjustments. Statistical analyses of multiple cytokine arrays and Western blots were performed using Prism version 5 graphing software (GraphPad Software). Throughout, significance levels are indicated as follows: *p* < 0.05, * in figures; *p* < 0.01, ** in figures; and *p* < 0.001, *** in figures.

## Results

### Monocyte accumulation after RD

**Fig. 1:**
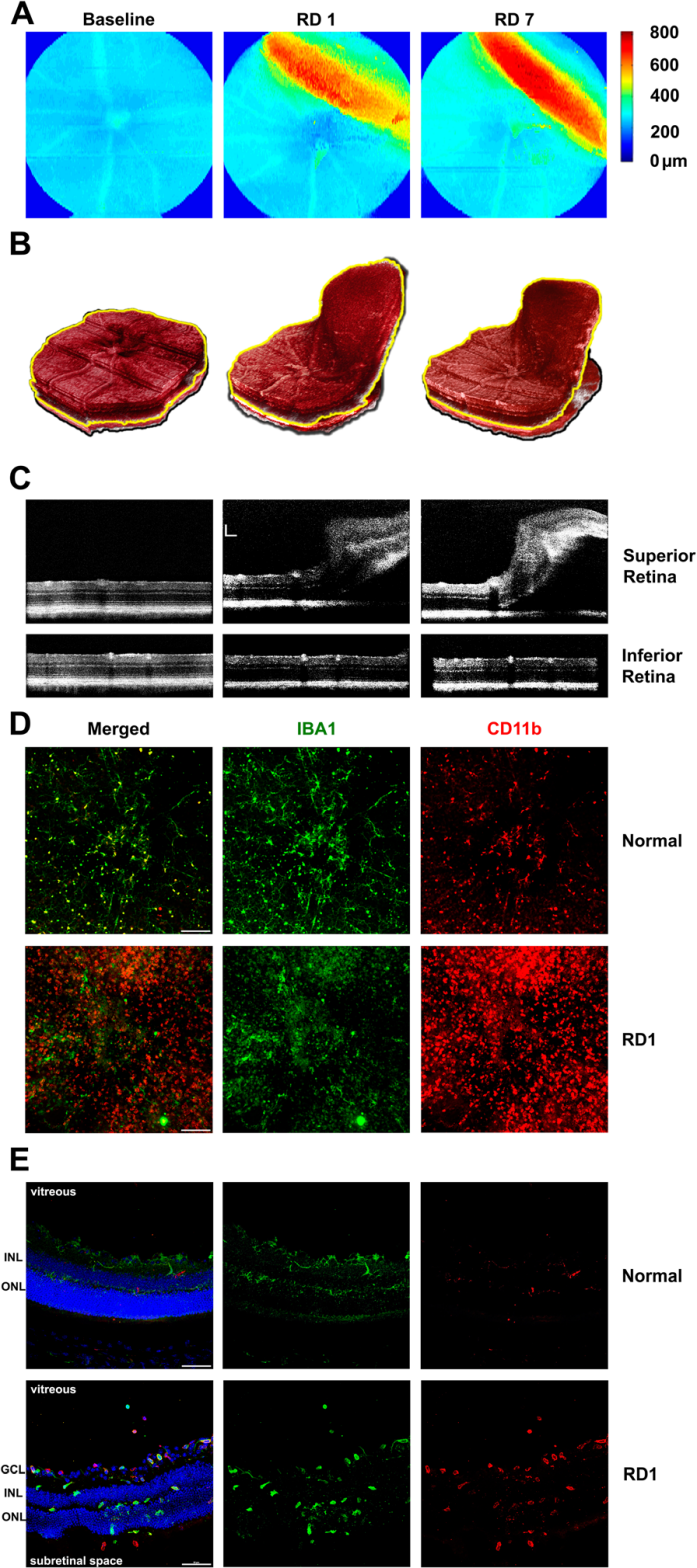
OCT imaging and immunohistochemistry labeling after retinal detachment (RD). **a** Retinal thickness fundus maps and **b** corresponding segmented retinal volumes revealed displacement of the superior retina into the vitreous cavity. In b, location of inner segment/outer segment border is marked by yellow line. **c** Individual OCT b-scans extracted from volumes in b demonstrated successful RD without any retinal holes. Scale bar = 200 μm. **d** Retina flat mounts (bar = 100 μm) and **e** cryosections (bar = 50 μm) were used to investigate the spatial distribution of bone marrow-derived monocytes (CD11b^high^, red round cells) and resident retinal microglia (Iba1^high^, green cells) after RD. In normal retina, there were no monocytes; resident microglia exhibited a resting, ramified-shape, and they were initially confined to ganglia and plexiform layers. After the first day of detachment, an increased number of monocytes infiltrated into untreated RD retina; microglia were activated and showed amoeboid morphology. Recruited monocytes floated in the vitreous space, infiltrated the ganglia layer (GCL), inner plexiform layer, and inner nuclear layer (INL). Both nuclear layers were defined using DAPI (blue). Activated microglia also became phagocytic, migrated into the outer nuclear layer, and engulfed photoreceptor cell bodies. Choroid-derived macrophages accumulated in the subretinal space

Sodium hyaluronate was injected into the subretinal space from scleral side, which avoided passage of the needle through the vitreous and puncturing the retina to induce RD. Using optical coherence tomography (OCT), we monitored the location and extent of the retinal detachment (Fig. 1a-c). RD caused a transient, acute retinal edema that resolved by seven days, consistent with a previous study [16]. None of the IL6 perturbations (IL6 KO, injection of anti-IL6Rα antibody or anti-gp130 antibody) caused retinal atrophy in the area of detachment (data of retinal thickness not shown). Overall, perturbing IL6 function did not per se cause dramatic retina atrophy, and thus are useful tools for assessing the role of IL6 in the immune response to RD.

To assess the cellular changes in early intraocular inflammation, we prepared retinal flat mounts and cryosections from normal control eyes and RD eyes at one day following detachment. Ramified microglia were distributed homogeneously across the retinal surface in normal control flat mount (Iba1^high^, Fig. 1d, shown in green) and had the expected normal ramified appearance in ganglion cell layer and plexiform layers in cryosections (Fig. 1e). One day after RD, there was a dramatic appearance of small round monocytes (CD11b^high^, Fig. 1d, shown in red). Cryosections further confirmed CD11b^high^ monocytes infiltrating from the vitreous to inner retina (Additional file 3: Figure S1B), as well as ameboid Iba1^high^ cells in the outer nuclear layer and ameboid CD11b^high^ macrophages in the subretinal space (Fig. 1e).

### Differential functions for gp130 and IL6Rα in monocyte recruitment and differentiation

**Fig. 2:**
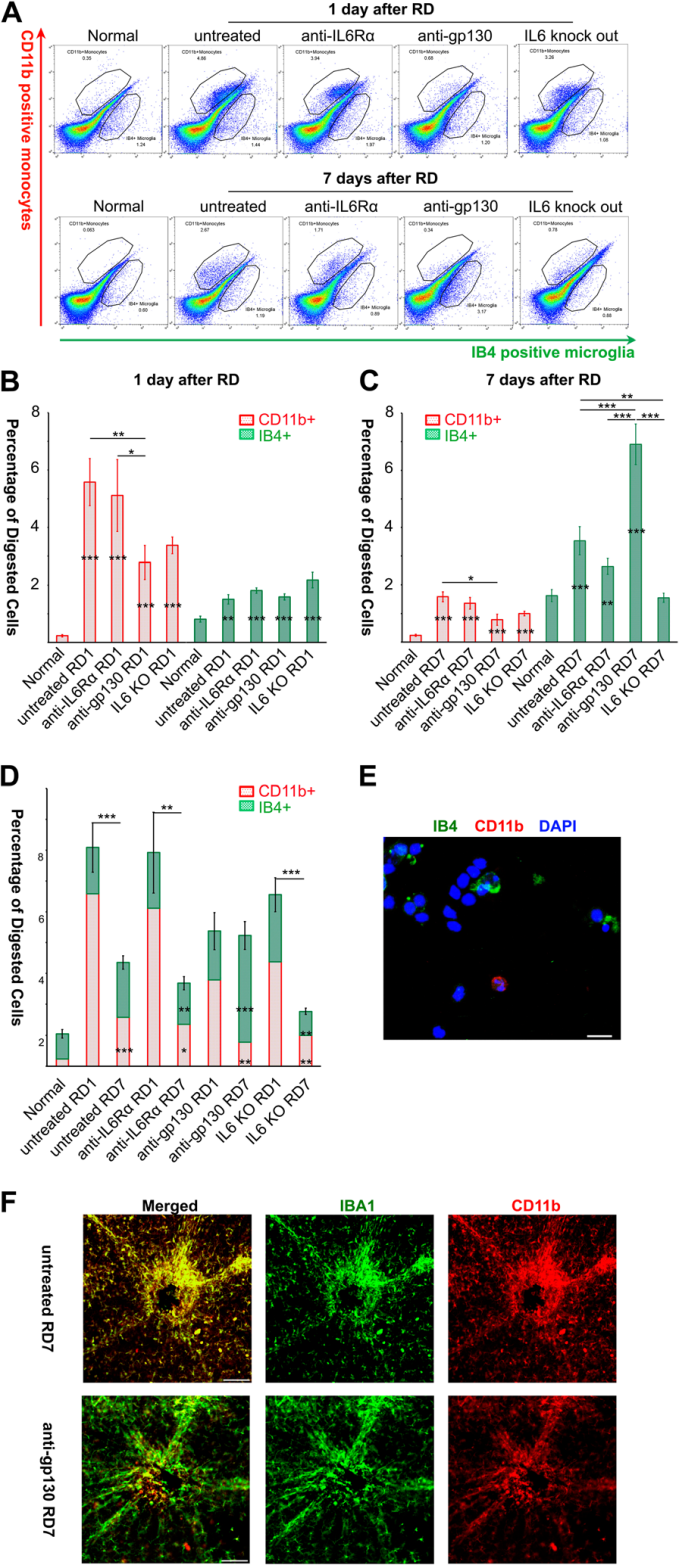
Quantification of recruited monocytes and resident microglia with Flow Cytometry. **a** Representative dot plots for CD11b^high^ IB4 ^low^ monocytes (top left gate) and CD11b^low^ IB4^high^ resident microglia (lower right gate) in 5 groups. **b** After one day, there was significant monocyte infiltration in all RD groups, as well as an increased number of microglia (normal = 8 mice; anti-IL6 Rα group = 16 mice; IL6 KO group = 6 mice). Compared with untreated RD1 retinas (n = 16 mice), only anti-gp130 treated retinas (n = 13 mice) had fewer monocytes recruited (**p < 0.01). **c** After seven days, anti-gp130 still (n = 8 mice) had fewer monocytes compared with untreated RD7 retinas (n = 14 mice, *p < 0.05). The other three RD7 groups sustained substantial monocyte infiltration (untreated RD7 ***p < 0.001; anti-IL6 Rα =13 mice, ***p < 0.001, IL6 knockout =6 mice ***p < 0.001). Moreover, the number of resident microglia increased significantly in the untreated (***p < 0.001), anti-IL6 Rα(**p < 0.01) and anti-gp130 RD7 retinas (***p < 0.001). Interestingly, anti-gp130 seemed to boost microglia numbers (compared with untreated RD7, ***p < 0.001), while IL6 knockout inhibited this rise in microglia numbers (**p < 0.01). **d** Total numbers of microglia and monocytes in each group. In the untreated and anti-IL6 Rα RD groups, total immune cells fell after seven days (***p < 0.001 and ** p < 0.01), mainly due to fewer monocytes (***p < 0.001 and * p < 0.05). RD retinas with anti-gp130 treatment had no significant change in total immune cell numbers, but replenished more microglia (***p < 0.001). In IL6 knockout mice, total immunes cells decreased sharply (***p < 0.001), and had the fewest microglia numbers (**p < 0.01). **e** Fluorescent microscope image of dissociated retinal cells used prior to flow cytometry. Retinas were digested, CD11b^high^ monocytes (red round cells) and IB4^high^ microglia (green cells) were isolated, nuclei were labeled with DAPI (bar = 10 μm). **f** Flat mounts (bar = 100 μm) showed that gp130 blocking antibody was associated with increased numbers of iba1^high^ cells, suggesting it promoted the differentiation of monocytes (CD11b^high^, red) into microglia-like macrophages (green) at seven days after RD as evidence by immunohistochemistry co-labeling

We quantified monocyte and microglia populations after RD using flow cytometry of dissociated retinal cells (Fig. 2a-e). As expected, in normal control retinas the average number of CD11b^high^ monocytes was far less than the number of IB4^high^ microglia. One day after RD, the number of monocytes increased more than 20-fold, and the number of microglia nearly doubled (Fig. 2b). The total number of immune cells decreased seven days after RD, primarily due to a relative decrease in CD11b^high^ monocytes (Fig. 2d; compare RD1 with RD7), though monocyte numbers remained elevated above normal in all groups (*p* < 0.001; Fig. 2c, red). We interpret these results to mean that the large influx of infiltrating monocytes seen on the first day was not sustained, and that the early-entering monocytes differentiated into microglia, as evidenced by increased numbers of cells co-stained for CD11b and Iba1 (Fig. 2f).

Because IL6 increases in the ocular fluids of RD human patients [9], we examined the extent of monocyte infiltration following RD in the absence of IL6 signaling. In mice lacking IL6 expression entirely (IL6 KO mice), the number of CD11b + cells one day after RD was reduced between IL6 KO RD mice and untreated RD (wild-type, *n* = 16 mice) mice (Fig. 2a-d), but this reduction was not statistically significant (*p* = 0.926; *n* = 6 mice). However, there was a significant reduction in IB4+ cells seven days after detachment in IL6 KO than wild-type RD retinas (Fig. 2c, green, *p* < 0.01; *n* = 6), suggesting that IL6 helps to direct monocyte-to-macrophage differentiation following extravasation.

To examine the consequences of blocking IL6 signaling in wild-type mice, we performed intravitreal injection of IL6 receptor antibodies immediately following retinal detachment. A blocking antibody against the IL6 receptor alpha subunit (anti-IL6Rα) had no effect on the numbers of CD11b + or IB4+ cells one day (Fig. 2b, *n* = 16 mice) nor seven days (Fig. 2c, *n* = 13 mice) after detachment. In contrast, intravitreal injection of a blocking antibody against the auxiliary subunit β (gp130), which is shared by several other cytokines, lead to a significant reduction in CD11b + monocytes compared with untreated RD samples (*p* < 0.01) and RD samples treated with anti-IL6Rα antibody (*p* < 0.05) at both one day (Fig. 2b, *n* = 13 mice) and seven days after detachment (Fig. 2c, *n* = 8 mice, *p* < 0.05 vs untreated RD7 samples, *n* = 14 mice). Intravitreal injection of an isotype control antibody (*n* = 3, *p* < 0.01) or PBS vehicle alone (*n* = 4, *p* < 0.001) immediately after retinal detachment did not reduce monocyte infiltration like injection of the anti-gp130 antibody (*n* = 4; Additional file 3: Figure S1c)

An even more pronounced effect of anti-gp130 treatment was evident on the population of IB4+ cells, resulting in a doubling of the number of microglia relative to untreated RD seven days after detachment (*p* < 0.001; Fig. 2c).

Together, these results suggest that after RD, circulating monocytes infiltrate into retina rapidly in a manner that does not require IL6, but gp130 signaling – presumably through the action of some other cytokine – promotes monocyte infiltration and subsequent differentiation of monocytes.

### Limited cytokine storm in the retina after RD

To identify cytokine changes induced by the retinal detachment, we subjected homogenates of normal and detached retinas to screening using proteomic arrays (Table 1). Most cytokines/chemokines showed no change one day following retinal detachment. Three showed a significant increase (*p* < 0.05) relative to normal control retinas: CCL2 (monocyte chemotactic protein 1), TIMP-1 (tissue inhibitor of metalloproteinase 1), and sICAM-1 (soluble intercellular adhesion molecule-1). CCL2 increased 15-fold in untreated RD1 retinas compared to normal control retinas (*p* < 0.001), and was reduced by all IL6 perturbations, with the IL6 KO being indistinguishable from normal control retina (no detachment; Table 1). This suggests that retinal CCL2 expression is downstream of IL6. Likewise, TIMP-1, which is secreted by monocytes/macrophages, was significantly increased in untreated RD1 retinas compared to normal control retinas (*p* < 0.05). In IL6 KO mice, TIMP-1 expression was dramatically downregulated in retina one day after RD (*p* < 0.05) Neither anti-IL6Rα nor anti-gp130 antibody treatment with RD altered TIMP-1 expression. Finally, sICAM-1, a circulating form of ICAM-1 (intercellular adhesion molecule 1) that is highly expressed in the vitreous of PVR patients [17], showed sustained, increased sICAM-1 expression for up to seven days after detachment (*p* < 0.01). The expression of sICAM-1 was not affected by any of the treatment groups (IL6 KO, anti-IL6α, or anti-gp130), suggesting that sustained retinal expression of sICAM-1 reflects the intrinsic physical trauma, rather than the immune response per se.

**Table 1:** Normalized Cytokines and Chemokines Expression in Detached Retinas

RD 1 day (Mean ± SD)				RD 7 days (Mean ± SD)				
Untreated (n = 6)	Anti-IL6Rα (n = 6)	Anti-gp130 (n = 6)	IL6 KO (n = 6)	Untreated (n = 8)		Anti-IL6Rα (n = 6)	Anti-gp130 (n = 8)	IL6 KO (n = 6)
CXCL1	1.2 ± 0.7	1.2 ± 0.3	0.9 ± 0.3	0.7 ± 0.1	0.5 ± 0.1	0.3 ± 0.2	0.6 ± 0.4	0.6 ± 0.3
CXCL2	1.3 ± 1.3	0.6 ± 0.1	0.3 ± 0.1	0.1 ± 0.0	0.2 ± 0.2	0.2 ± 0.1	0.3 ± 0.2	0.2 ± 0.1
CXCL9	0.6 ± 0.4	1.3 ± 0.5	0.6 ± 0.5	0.3 ± 0.1	0.4 ± 0.4	0.4 ± 0.2	0.4 ± 0.2	0.2 ± 0.1
CXCL10	1.2 ± 0.5	1.7 ± 0.4	1.1 ± 0.3	0.7 ± 0.2	0.7 ± 0.3	0.9 ± 0.9	0.7 ± 0.5	0.4 ± 0.2
CXCL11	0.6 ± 0.3	1.0 ± 0.4	0.6 ± 0.2	0.4 ± 0.1	0.4 ± 0.2	0.3 ± 0.1	0.4 ± 0.3	0.3 ± 0.2
CXCL12	0.8 ± 0.1	0.9 ± 0.3	0.9 ± 0.2	0.7 ± 0.2	0.6 ± 0.3	0.5 ± 0.2	0.7 ± 0.3	0.9 ± 0.1
CXCL13	1.0 ± 0.5	1.0 ± 0.2	1.0 ± 0.1	0.8 ± 0.2	0.5 ± 0.1	0.4 ± 0.1	0.6 ± 0.4	0.5 ± 0.2
CCL1	0.6 ± 0.3	0.8 ± 0.2	0.8 ± 0.1	1.0 ± 0.1	0.5 ± 0.2	0.4 ± 0.1	0.6 ± 0.3	0.6 ± 0.0
**CCL2**	**15.1** ± **6.9**	**4.4** ± 0.7*	**1.7** ± 1.0**	**1.2** ± 0.4**	**0.7** ± **0.4**	**1.0** ± **0.8**	**0.6** ± **0.3**	**0.5** ± **0.1**
CCL3	1.5 ± 1.3	1.5 ± 0.6	0.7 ± 0.5	0.3 ± 0.1	0.3 ± 0.2	0.3 ± 0.1	0.4 ± 0.2	0.4 ± 0.2
CCL4	0.8 ± 0.2	0.7 ± 0.1	0.7 ± 0.1	0.8 ± 0.1	0.5 ± 0.2	0.3 ± 0.1	0.6 ± 0.3	0.8 ± 0.1
CCL5	1.7 ± 1.4	2.1 ± 0.7	1.1 ± 0.5	0.9 ± 0.2	0.6 ± 0.1	0.6 ± 0.1	0.5 ± 0.1	0.7 ± 0.1
CCL11	0.6 ± 0.2	0.8 ± 0.1	0.8 ± 0.2	0.5 ± 0.1	0.3 ± 0.1	0.3 ± 0.0	0.5 ± 0.3	0.5 ± 0.1
CCL12	1.1 ± 0.5	1.3 ± 0.3	1.0 ± 0.5	0.7 ± 0.1	0.6 ± 0.1	0.5 ± 0.2	0.6 ± 0.3	0.6 ± 0.4
CCL17	0.8 ± 0.4	0.8 ± 0.1	1.2 ± 0.3	1.4 ± 0.2	0.5 ± 0.2	0.3 ± 0.0	0.6 ± 0.4	0.5 ± 0.4
IL-1α	0.8 ± 0.1	0.7 ± 0.1	1.0 ± 0.1	0.7 ± 0.1	0.5 ± 0.2	0.5 ± 0.4	0.8 ± 0.3	1.0 ± 0.1
IL-1β	0.7 ± 0.4	1.0 ± 0.5	0.6 ± 0.4	0.2 ± 0.1	0.4 ± 0.2	0.3 ± 0.1	0.3 ± 0.1	0.3 ± 0.1
IL-1ra	1.9 ± 1.9	1.5 ± 0.2	0.7 ± 0.5	0.5 ± 0.2	0.6 ± 0.2	0.7 ± 0.4	0.6 ± 0.2	0.6 ± 0.1
IL2	0.6 ± 0.1	0.6 ± 0.1	0.7 ± 0.1	0.7 ± 0.2	0.6 ± 0.3	0.4 ± 0.3	0.5 ± 0.2	0.7 ± 0.1
IL-3	0.8 ± 0.3	1.5 ± 0.1	0.8 ± 0.4	0.6 ± 0.2	0.4 ± 0.2	0.3 ± 0.2	0.5 ± 0.4	0.4 ± 0.4
IL-4	0.8 ± 0.5	1.2 ± 0.3	0.6 ± 0.3	0.3 ± 0.1	0.3 ± 0.3	0.3 ± 0.2	0.3 ± 0.2	0.2 ± 0.2
IL-5	0.5 ± 0.3	0.8 ± 0.2	0.7 ± 0.2	0.7 ± 0.1	0.5 ± 0.2	0.3 ± 0.2	0.4 ± 0.3	0.4 ± 0.2
**IL**-**6**	**0.6** ± **0.2**	**0.8** ± **0.1**	**0.8** ± **0.2**	**0.8** ± **0.2**	**0.5** ± **0.1**	**0.3** ± **0.1**	**0.7** ± **0.5**	**0.6** ± **0.5**
IL-7	0.6 ± 0.4	0.9 ± 0.3	0.6 ± 0.2	0.2 ± 0.1	0.2 ± 0.1	0.2 ± 0.1	0.4 ± 0.3	0.3 ± 0.1
IL-10	0.6 ± 0.2	0.7 ± 0.0	0.6 ± 0.1	0.8 ± 0.2	0.3 ± 0.0	0.3 ± 0.0	0.4 ± 0.1	0.5 ± 0.0
IL-13	0.7 ± 0.5	1.4 ± 0.6	0.6 ± 0.4	0.2 ± 0.0	0.3 ± 0.2	0.3 ± 0.1	0.3 ± 0.2	0.3 ± 0.0
IL-12 p70	0.5 ± 0.1	0.7 ± 0.1	0.7 ± 0.2	0.4 ± 0.1	0.4 ± 0.1	0.3 ± 0.1	0.5 ± 0.4	0.5 ± 0.2
IL-16	0.9 ± 0.6	0.6 ± 0.0	0.7 ± 0.1	0.6 ± 0.1	0.4 ± 0.1	0.3 ± 0.0	0.7 ± 0.5	0.6 ± 0.4
IL-17	0.4 ± 0.3	0.6 ± 0.2	0.4 ± 0.2	0.2 ± 0.1	0.2 ± 0.2	0.2 ± 0.1	0.3 ± 0.3	0.3 ± 0.1
IL-23	0.6 ± 0.2	0.8 ± 0.1	0.6 ± 0.3	0.4 ± 0.1	0.4 ± 0.1	0.4 ± 0.1	0.5 ± 0.2	0.5 ± 0.1
**IL**-**27**	**0.8** ± **0.5**	**1.6** ± **0.5**	**0.6** ± **0.7**	**0.2** ± **0.1**	**0.3** ± **0.2**	**0.4** ± **0.1**	**0.4** ± **0.2**	**0.3** ± **0.2**
G-CSF	0.6 ± 0.4	1.1 ± 0.3	0.9 ± 0.3	0.6 ± 0.0	0.4 ± 0.0	0.3 ± 0.1	0.5 ± 0.2	0.4 ± 0.1
GM-CSF	0.6 ± 0.3	0.9 ± 0.2	1.0 ± 0.2	0.9 ± 0.1	0.5 ± 0.2	0.3 ± 0.0	0.7 ± 0.3	0.6 ± 0.3
M-CSF	0.9 ± 0.5	1.6 ± 0.3	0.9 ± 0.8	0.3 ± 0.1	0.5 ± 0.3	0.5 ± 0.3	0.6 ± 0.4	0.4 ± 0.4
**sICAM**-**1**	**2.3** ± **0.7**	**2.3** ± **0.3**	**2.2** ± **0.3**	**1.8** ± **1.0**	**2.7** ± **0.1**	**2.6** ± **0.5**	**2.3** ± **0.4**	**2.0** ± **0.6**
IFN-γ	0.7 ± 0.4	1.3 ± 0.8	0.7 ± 0.4	0.3 ± 0.1	0.3 ± 0.1	0.3 ± 0.1	0.4 ± 0.2	0.6 ± 0.1
**TIMP**-**1**	**3.9** ± **3.9**	**3.6** ± **1.1**	**1.3** ± **1.5**	**0.5** ± 0.2*	**1.2** ± **1.6**	**1.6** ± **1.9**	**0.9** ± **0.7**	**0.6** ± **0.7**
TNF-a	0.6 ± 0.3	0.8 ± 0.2	0.9 ± 0.2	1.1 ± 0.0	0.6 ± 0.3	0.5 ± 0.2	0.8 ± 0.3	0.9 ± 0.1
TREM-1	1.3 ± 0.7	2.1 ± 0.6	0.9 ± 0.6	0.5 ± 0.0	0.5 ± 0.3	0.5 ± 0.2	0.5 ± 0.2	0.4 ± 0.1
C5/C5a	0.6 ± 0.4	0.8 ± 0.2	0.5 ± 0.3	0.4 ± 0.1	0.2 ± 0.1	0.2 ± 0.1	0.4 ± 0.3	0.2 ± 0.3

Fluorescence values of multiple arrays were normalized to normal control (not detachment) retinas, shown in ratios and statistically compared with **untreated RD retina**. *p < 0.05, **p < 0.01, ***p < 0.001

**Fig. 3:**
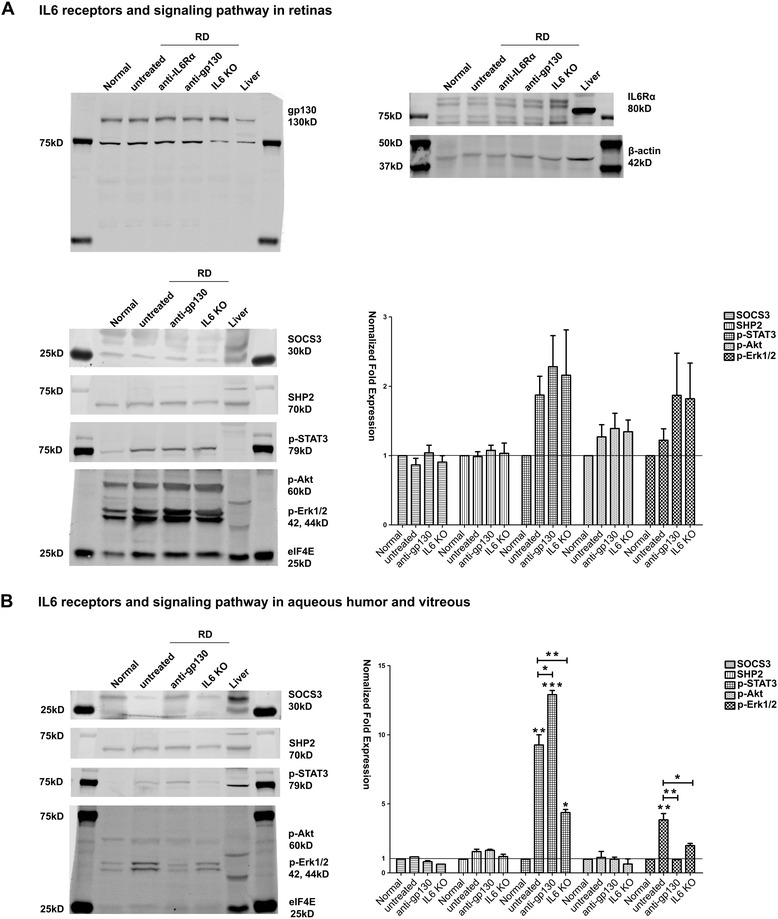
Protein expression of IL6/gp130 signaling pathway in mice one day after RD. **a** In retinas (n = 6 mice for each group), gp130 expression increased non-significantly after RD and was consistently expressed in all treatment groups. In contrast, membrane-bound IL6Rα was undetectable (note very high IL6Rα expression in liver). The key regulatory proteins of IL6 signaling, SOCS3, SHP2, p-STAT3, p-Akt and p-Erk1/2, all showed no significant changes in the retina one day after RD (one-way ANOVA, Tukey’s test). **b** In aqueous/vitreous humor samples (n = 10 mice for each group), p-STAT3 was up-regulated 9-fold (compared with normal, ** p < 0.01), anti-gp130 further promoted this phosphorylation (13-fold higher than normal, *** p < 0.001; compared with untreated RD1 group, * p < 0.05); while IL6 KO weakened this upregulation (4-fold, compared with normal, * p < 0.05; compared with untreated RD1 group, ** p < 0.01). p-Erk1/2 was also up-regulated after RD (4-fold, compared with normal, ** p < 0.01), while anti-gp130 (0.96 fold, compared with untreated RD1 group, ** p < 0.01) and IL6 KO (1.98 fold, compared with untreated RD1 group, * p < 0.05) both inhibited p-Erk1/2 activation. Note: the intensities of SOCS3, SHP2, p-STAT3, p-Akt and p-Erk1/2 bands in all 3 detached groups were normalized to their intensities of normal group, and then the normalized folds were analyzed using one-way ANOVA followed by Tukey-Kramer adjustments

Surprisingly, multiple cytokine arrays showed very low IL6 retinal expression in both normal and detached conditions (Additional file 4: Figure S2a). In addition, IL6Rα, which is a plasma membrane protein, was undetectable in retinal samples via Western blot and immunohistochemistry (Additional file 4: Figure S2b-d). Retinal gp130 expression was detectable (Additional file 3: Figure S1a), but did not change after RD in any groups (Fig. 3a). Furthermore, there were no significant changes in the retinal expression of IL6 downstream targets (Fig. 3a), including SOCS3, SHP2, p-STAT3, p-Akt and p-Erk1/2. IL27, which is highly expressed in mouse retina and also utilizes the gp130 receptor, also did not significantly change after RD (Table 1 and Additional file 4: Figure S2a). These results suggest that there is little IL6 or gp130 signaling occurring in the retina itself after detachment and instead that the consequences of IL6 KO and IL6 receptor antibody blockades acted directly on the infiltrating cells.

### IL6/IL6 Rα signaling is more active in the aqueous/vitreous humor than in the retina

**Fig. 4:**
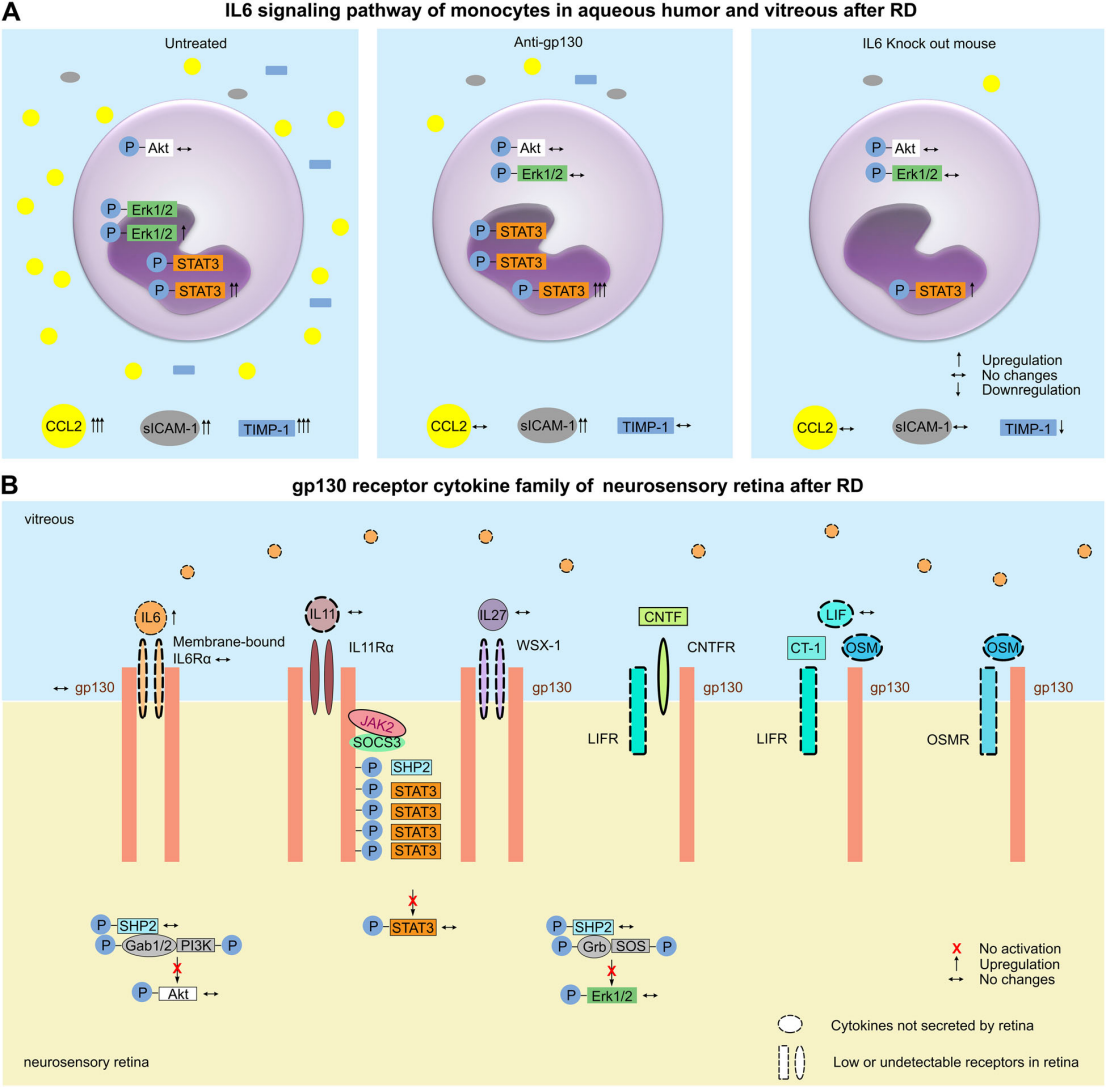
Model summary of intraocular IL6 and gp130 signaling. **a** One day after RD, retina secretes CCL2, sICAM-1 and TIMP-1. Bone marrow derived-monocytes are recruited into the vitreous. Active monocytes upregulate p-STAT3 and p-Erk1/2. However, anti-gp130 treatment inhibits CCL2 secretion and monocyte recruitment, by strengthening p-STAT3 and downregulating p-Erk1/2 in vitreous monocytes after RD. IL6 knockout mice inhibit CCL2 and TIMP-1 secretion, weaken p-STAT3 and p-Erk1/2 activation after RD. **b** Seven cytokines signal via gp130, but most of the them and their co-receptors have very weak or undetectable expression (shown by dotted line) in mouse retina, including IL6 and receptor IL6Rα. In the retina, gp130 only signals through IL11/IL11Rα (shown by solid line) but IL11 is not changed after RD. This can explain why there was no IL6/IL6Rα protein expression in retina and why there were no significant changes of gp130, p-STAT3, p-Akt and p-Erk1/2 in retina after RD in our study. Despite high IL6 in vitreous (secreted by monocytes), the retinal IL6/IL6Rα signaling is silent. Therefore, anti-gp130, not anti-IL6 or anti-IL6Rα, may be beneficial to retina, by intervening early inflammation after RD

Retinal IL6/IL6Rα was undetectable via cytokines arrays, Western blot and immunohistochemistry in both normal control and RD retinas (Additional file 4: Figure S2a-d). Comparable interrogation of aqueous/vitreous humor by cytokine expression profiling was not possible because of the very small volumes and relative low target protein content. However, Western blot analysis of aqueous humor and vitreous samples showed an increase in IL6 downstream effectors (Fig. 3b), p-STAT3 (9.26 fold, *p* < 0.01) and p-Erk1/2 (3.84 fold, *p* < 0.01) after retinal detachment (RD1). Anti-gp130 treatment coincident with detachment increased STAT3 phosphorylation (12.9 fold, *p* < 0.001) above that of the untreated RD1 group (*p* < 0.05). Similarly, the IL6 KO RD1 samples showed a 4.37-fold upregulation of p-STAT3 (*p* < 0.05) over the untreated RD1 group (*p* < 0.01). Both anti-gp130 treated (*p* < 0.01) and loss of IL6 expression (IL6 KO mice; *p* < 0.05) prevented the RD1-induced increase in p-ERK1/2. Together (Fig. 4), these results suggest that IL6 signaling in the aqueous/vitreous occurs via gp130, and that anti-gp130 treatment targeted monocytes in the vitreous, not in the retina, causing an upregulation of p-STAT3 and an inhibition of p-Erk1/2 in those cells.

## Discussion

### Vitreal monocytes as the predominant source of IL6 following RD

High IL6 levels have been correlated with the severity of RD and the incidence of PVR complications [8]. In our experiments, retinal IL6 protein expression was undetectable following retinal detachment (Table 1; Additional file 4: Figure S2a), which is consistent with RNAseq data showing low IL6 gene expression [18]. These results suggest that although high levels of IL6 are evident in human samples of aqueous humor [7], vitreous [9] and subretinal fluid [7–9], it is apparently not secreted by the retina, but rather by invasive cells [10] in the vitreous.

The identity of the cells secreting IL6 has been ambiguous. In our mouse model of RD, hemi-retinal detachment was induced without any retinal holes, obviating the migration of subretinal macrophages and RPE cells into vitreous and retina surface and thus removing these cells as likely sources of IL6. Instead, our study shows that bone marrow-derived monocytes rapidly infiltrate the vitreous (Additional file 3: Figure S1b) within one day after detachment, and likely are the source of IL6 rather than merely the cells responding to IL6.

Clinical studies have demonstrated that epiretinal membranes (ERM) from PVR patients had the greatest density of immune cells compared with those with successful retinal detachment repair, those with proliferative diabetic retinopathy, or those with idiopathic ERM patients [19]. Thus, inhibiting monocyte extravasation and their IL6 secretion in the early stages of RD may reduce inflammation and the risk of PVR development in RD patients. Interestingly, our Western blotting results failed to detect membrane-bound IL6Rα protein both in the retina (Fig. 3a) and aqueous/vitreous humor (Additional file 4: Figure S2 b,c) before or after RD, consistent with previous reports of very low IL6Rα gene expression in mouse retina [18] but higher levels of soluble IL6Rα in vitreous humor [20] of PVR patients. This suggests that the IL6 secreted by monocytes further impinges upon monocytes or blood vessels via IL6 trans-signaling, rather than retinal neurons via classic IL6 signaling. Given the low IL6Rα expression in the retina, it is not too surprising that there were also no significant changes in key regulatory proteins downstream of IL6 signaling in the retina, including p-STAT3, p-Akt and p-Erk1/2 (Fig. 3a). In contrast, aqueous/vitreous humor samples from RD mice showed strong upregulation of p-STAT3 (9-fold) and p-Erk1/2 (4-fold). These results suggest the view that the aqueous and vitreous humor, rather than the retina or subretinal space, are the key compartments where IL6/gp130 signaling is regulated in RD.

### The role of the retina in retinal detachment

Our cytokine array results revealed a dramatic (15-fold) increase in retinal CCL2 levels within one day of RD (Table 1). CCL2, also known as monocyte chemoattractant protein 1 (MCP1), is the key chemokine regulating migration and infiltration of monocytes/macrophages [21]. In the rat RD model, CCL2 likewise increased rapidly and subsided after three days [22], driving influx of CCR2+ monocytes [23]. The primary intraocular source of CCL2 after RD is thought to be Müller glia [24, 25], and CCL2 blockade greatly reduced macrophage/microglia infiltration and photoreceptor apoptosis [24]. Strong correlation between IL6 and CCL2 levels has been found in most human vitreoretinal diseases [26–28], consistent with both of these chemokines being temporally associated with monocyte infiltration. However, we also found that loss of IL6 or blockade of each of its two receptors all inhibited the RD induced increase in retinal CCL2 levels (Table 1). Since neither IL6 nor IL6Rα are expressed at detectable levels in the retina, our results suggest that gp130 mechanistically lies between monocyte-secreted IL6 in the vitreous and the induction of CCL2 expression in the Müller glia.

We also observed a ~4-fold increase in the expression of retinal TIMP-1 within one day of RD (Table 1, *p* < 0.05). The tissue inhibitors of metalloproteinases (TIMPs) are specific inhibitors of the Matrix metalloproteinases (MMPs). Like CCL2, TIMP-1 levels also correlate significantly with IL6 levels in PVR patients [9], and are found in vitreous and subretinal fluid of RD patients in a manner correlated with the extent of the detachment [9, 28]. In previous mouse studies, TIMP-1 was upregulated for two weeks following RD [29], suggesting a sustained response to damage or an involvement in retinal healing. In our studies, blocking IL6 receptors did not affect TIMP-1 expression one day after RD (*p* > 0.05), though complete loss of IL6 (IL6 KO) drastically reduced TIMP-1 expression (Table 1). We infer that high IL6 levels promote early stage M1 macrophage polarization, while anti-IL6 may promote M2 macrophage polarization, the latter of which downregulates TIMP-1 expression [30] in the later stage of inflammation.

### Good or Bad? The role of IL6 signaling depends on the gp130 cytokine family

In our model of retinal detachment, IL6 KO mice did not show any fewer infiltrated monocytes after RD (Fig. 2b, c). This suggests that IL6 per se is not the master cytokine initiating inflammation for the retina after detachment. Thus, RD patients are unlikely to benefit from humanized monoclonal antibody (mAb) against IL6 only.

Blocking the downstream consequences of IL6, however, proved to have interesting and profound consequences for monocyte infiltration. A monoclonal blocking antibody against gp130 reduced monocyte recruitment (Fig. 2a), seemed to promote monocyte differentiation into microglia-like macrophages (Fig. 2b-d), and reduced retinal CCL2 secretion (Table 1). Anti-gp130 therapy also upregulated p-STAT3 and downregulated p-Erk1/2 in vitreous samples after RD (Fig. 3b). Because p-STAT3 upregulation and p-Erk1/2 inhibition have protective effects for photoreceptors [31] and RPE survival [32], these findings suggest that the anti-gp130 antibody is beneficial to detached retina. Similar results in a model of experimental colitis using gp130 (757 F/F) mice [33] supports the notion that gp130 is a pivotal molecule acting downstream of IL6 to more specifically control macrophage fate and tissue repair. Notably, these consequences of blocking downstream IL6 signaling was specific for gp130; blocking the canonical IL6 receptor, IL6Rα, had no effect on monocyte infiltration and the cytokine storm.

### Working model of distinct IL6 and gp130 signaling at the retina-vitreous interface

There are seven known cytokines that signal via gp130 (Fig. 4): IL6, IL11, IL27, CT-1, LIF, CNTF, and OSM. Gene sequencing of the gp130 cytokine family in mouse retina has shown [18] that there is very low gene expression of IL6, IL11, LIF or OSM (Fig. 4b, shown by dotted line) in retinal photoreceptors, horizontal cells, bipolar cells, amacrine cells, ganglion cell layer, and microglia. This means within the gp130 cytokine family, retina only has the intrinsic capacity for IL27, CT-1, and CNTF signaling (Fig. 4b, shown by solid line); the other four cytokines are exogenous and must be secreted by other cells (e.g., IL6 secreted by infiltrated monocytes). Many of the co-receptors that dimerize with the gp130 receptor to support cytokine signaling are also weakly expressed in the retina (Fig. 4b, dotted lines). Genes of IL6Ra, IL27 receptor (WSX-1), LIFR, CNTFR and OSMR are expressed at undetectable levels [18]. Only IL11 receptor α1 (IL11Rα1) and gp130 genes are highly expressed [18]. A comprehensive review of the gp130 cytokine family in mouse and human retina before and after RD [8, 10, 25, 27, 34–73] is shown in Additional file 2: Table S2.

Thus, the only cytokine/receptor combination expressed in the retina to support gp130 signaling is via IL11/IL11Rα. We predict that the anti-gp130 antibody inhibited CCL2 release from Müller glia using this signaling pathway. However, because IL11 is not significantly upregulated in the subretinal fluid of PVR [8] patients or gliotic human retina [25], we infer that IL11 is not associated with PVR development. This model thus explains why there was weak retinal IL6/IL6Rα expression and why there were no significant changes in gp130, p-STAT3, p-Akt and p-Erk1/2 levels after RD: the weak signaling pathway of gp130 in retina is due to negligible expression of cytokines and receptors.

## Conclusions

In summary, we found that following retinal detachment, monocyte infiltration into the vitreous and vitreo-retinal interface is rapid, and sets off a cascade of downstream cytokine events that further escalates immune response, like retinal CCL2 expression. The infiltrated monocytes release IL6, which promotes signaling through gp130 to control macrophage polarization. In humans, a clinical study revealed that intravitreal injection of steroids failed to decrease IL6 secretion in RD patients [53]. Our results in the mouse suggest that a more specific, cytokine-targeted therapy for RD patients may be more effective. So far, there are several FDA-approved monoclonal antibodies against IL6 signaling, including Clazakizumab and Olokizumab (anti-IL6) and Tocilizumab (anti-IL6Rα), yet none of them are likely to reduce RD-associated inflammation based on our results. In contrast, anti-gp130 may be a promising therapeutic strategy reduce early inflammation after RD when immediate re-attachment is not possible.

## Additional files

Additional file 1 Table S1. Antibodies used in the current study. (PDF 237 kb)

Additional file 2 Table S2. Ocular Expression of the gp130 Receptor Cytokine Family in Normal and Detached Retina of Mouse and Human. (PDF 219 kb)

Additional file 3 Figure S1. gp130 expression following acute retinal detachment (RD) and isotype control experiment. (A) The gp130 expression (red) and Müller cells (CRALBP, green) in normal and detached retinas (bar = 50 μm). Two nuclei layers were stained with DAPI (blue). Retinal gp130 expression was initially confined to outer nuclear layer (ONL) in normal retina, and extended into the inner nuclear layer and monocytes in vitreous humor after RD. (B) Recruited monocytes floated in the vitreous space one day after RD (bar = 10 μm). CD11b (green) and membrane-bound gp130 (red) co-stained in monocytes. Nuclei were stained with DAPI (blue). (C) Additional evidence to support the therapeutic effect of anti-gp130 antibody. Intravitreal injection of an isotype control antibody (*n* = 3, *p* < 0.01) or PBS vehicle alone (*n* = 4, *p* < 0.001) immediately after RD did not reduce monocyte infiltration like injection of the anti-gp130 antibody (*n* = 4). (JPG 9157 kb)

Additional file 4 Figure S2. IL6/IL6Rα expression before and after acute retinal detachment. (A) Multiple cytokines arrays showed very low IL6 expression in either normal or detached retinas. Most cytokines/chemokines showed no change one day following RD, while CCL2 and TIMP-1 are found to be strongly related to IL6 signaling. They both significantly increased in untreated RD1 retinas compared to normal retinas, while detached retinas of IL6 KO mice showed downregulation of CCL2 and TIMP-1. Interestingly, IL27, which shares the same receptor gp130 with IL6 signaling, did not significantly change after RD. IL27 expression seemed weaker in IL6 KO retinas after RD, but didn’t have significant difference (one-way ANOVA, Tukey-Kramer adjustments). (B) The membrane-bound IL6 receptor α (IL6R α) was undetectable in the retina via western blot, while the IL6 receptor β (gp130) has stable expression in normal and IL6 KO retinas. (C) One day after RD, the gp130 expression was detected and increased in aqueous humor/vitreous via western blot, while membrane-bound IL6R α was still undetectable. (D) We verified IL6Rα protein expression by immunohistochemistry in normal retina and detached retina using 3 different primary antibodies and isotype control. They all showed nonspecific binding and low expression of IL6Rα. (JPG 8267 kb)

## Abbreviations

Akt: Protein kinase B
CNTF: Ciliary neurotrophic factor
CT-1: Cardiotrophin-1
gp130: Glycoprotein 130, also known as IL6Rβ
ICAM-1: Intercellular adhesion molecule-1
IL11: Interleukin 11
IL27: Interleukin 27
IL6: Interleukin 6
IL6Rα: IL6 receptor α
KO: Knockout
LIF: Leukemia inhibitory factor
mAb: Monoclonal antibody
MCP-1/CCL2: Monocyte chemotactic protein 1
MMPs: Matrix metalloproteinases
OSM: Oncostatin M
p-Akt: Phospho-Akt
p-Erk1/2: Phospho-extracellular signal–regulated kinase1 and 2
p-STAT3: Phospho-Stat3
PVR: Proliferative vitreoretinopathy
RD: Retinal detachment
RPE: Retinal pigment epithelium
SHP2: Tyrosine phosphatase containing Src Homology 2 domains
SOCS3: Suppressor of cytokine signaling 3
STAT3: Signal transducer and activator of transcription 3
TIMP-1: Tissue inhibitor of metalloproteinase 1
WSX-1: IL27 receptor

## References

[1] Chen SN, Lian Ie B, Wei YJ (2016). Epidemiology and clinical characteristics of rhegmatogenous retinal detachment in Taiwan. Br J Ophthalmol.

[2] Shah V, Hall N, Goldacre MJ (2015). Retinal detachment in England: database studies of trends over time and geographical variation. Br J Ophthalmol.

[3] Hajari JN, Bjerrum SS, Christensen U, Kiilgaard JF, Bek T, La Cour M (2014). A nationwide study on the incidence of rhegmatogenous retinal detachment in Denmark, with emphasis on the risk of the fellow eye. Retina.

[4] Park SJ, Choi NK, Park KH, Woo SJ (2013). Five year nationwide incidence of rhegmatogenous retinal detachment requiring surgery in Korea. PLoS ONE.

[5] Daien V, Le Pape A, Heve D, Carriere I, Villain M (2015). Incidence, Risk Factors, and Impact of Age on Retinal Detachment after Cataract Surgery in France: A National Population Study. Ophthalmology.

[6] Tseng W, Cortez RT, Ramirez G, Stinnett S, Jaffe GJ (2004). Prevalence and risk factors for proliferative vitreoretinopathy in eyes with rhegmatogenous retinal detachment but no previous vitreoretinal surgery. Am J Ophthalmol.

[7] Hoerster R, Hermann MM, Rosentreter A, Muether PS, Kirchhof B, Fauser S (2013). Profibrotic cytokines in aqueous humour correlate with aqueous flare in patients with rhegmatogenous retinal detachment. Br J Ophthalmol.

[8] Ricker LJ, Kijlstra A, Kessels AG, De Jager W, Liem AT, Hendrikse F, La Heij EC (2011). Interleukin and growth factor levels in subretinal fluid in rhegmatogenous retinal detachment: a case–control study. PLoS ONE.

[9] Symeonidis C, Papakonstantinou E, Androudi S, Georgalas I, Rotsos T, Karakiulakis G, Diza E, Dimitrakos SA (2014). Comparison of interleukin-6 and matrix metalloproteinase expression in the subretinal fluid and the vitreous during proliferative vitreoretinopathy: correlations with extent, duration of RRD and PVR grade. Cytokine.

[10] El-Ghrably IA, Dua HS, Orr GM, Fischer D, Tighe PJ (2001). Intravitreal invading cells contribute to vitreal cytokine milieu in proliferative vitreoretinopathy. Br J Ophthalmol.

[11] Kopf M, Bachmann MF, Marsland BJ (2010). Averting inflammation by targeting the cytokine environment. Nat Rev Drug Discov.

[12] Matsumoto H, Miller JW, Vavvas DG. Retinal detachment model in rodents by subretinal injection of sodium hyaluronate. J Vis Exp. 2013 Sep 11;79. 10.3791/50660 PMC3864357 24056325

[13] Zhang P, Zam A, Jian Y, Wang X, Li Y, Lam KS, Burns ME, Sarunic MV, Pugh EN, Zawadzki RJ (2015). In vivo wide-field multispectral scanning laser ophthalmoscopy-optical coherence tomography mouse retinal imager: longitudinal imaging of ganglion cells, microglia, and Muller glia, and mapping of the mouse retinal and choroidal vasculature. J Biomed Opt.

[14] Zawadzki RJ, Fuller AR, Wiley DF, Hamann B, Choi SS, Werner JS (2007). Adaptation of a support vector machine algorithm for segmentation and visualization of retinal structures in volumetric optical coherence tomography data sets. J Biomed Opt.

[15] Legroux L, Pittet CL, Beauseigle D, Deblois G, Prat A, Arbour N (2015). An optimized method to process mouse CNS to simultaneously analyze neural cells and leukocytes by flow cytometry. J Neurosci Methods.

[16] Cebulla CM, Ruggeri M, Murray TG, Feuer WJ, Hernandez E (2010). Spectral domain optical coherence tomography in a murine retinal detachment model. Exp Eye Res.

[17] Limb GA, Chignell AH (1999). Vitreous levels of intercellular adhesion molecule 1 (ICAM-1) as a risk indicator of proliferative vitreoretinopathy. Br J Ophthalmol.

[18] Siegert S, Cabuy E, Scherf BG, Kohler H, Panda S, Le YZ, Fehling HJ, Gaidatzis D, Stadler MB, Roska B (2012). Transcriptional code and disease map for adult retinal cell types. Nat Neurosci.

[19] Oberstein SY, Byun J, Herrera D, Chapin EA, Fisher SK, Lewis GP (2011). Cell proliferation in human epiretinal membranes: characterization of cell types and correlation with disease condition and duration. Mol Vis.

[20] Yamamoto H, Hayashi H, Uchida H, Kato H, Oshima K (2003). Increased soluble interleukin-6 receptor in vitreous fluid of proliferative vitreoretinopathy. Curr Eye Res.

[21] Deshmane SL, Kremlev S, Amini S, Sawaya BE (2009). Monocyte chemoattractant protein-1 (MCP-1): an overview. J Interferon Cytokine Res.

[22] Nakazawa T, Matsubara A, Noda K, Hisatomi T, She H, Skondra D, Miyahara S, Sobrin L, Thomas KL, Chen DF (2006). Characterization of cytokine responses to retinal detachment in rats. Mol Vis.

[23] Mossanen JC, Krenkel O, Ergen C, Govaere O, Liepelt A, Puengel T, Heymann F, Kalthoff S, Lefebvre E, Eulberg D, et al. CCR2+ monocytes aggravate the early phase of acetaminophen induced acute liver injury. Hepatology. 2016. 10.1002/hep.28682 27302828

[24] Nakazawa T, Hisatomi T, Nakazawa C, Noda K, Maruyama K, She HC, Matsubara A, Miyahara S, Nakao S, Yin YQ (2007). Monocyte chemoattractant protein 1 mediates retinal detachment-induced photoreceptor apoptosis. Proc Natl Acad Sci U S A.

[25] Eastlake K, Banerjee PJ, Angbohang A, Charteris DG, Khaw PT, Limb GA (2016). Muller glia as an important source of cytokines and inflammatory factors present in the gliotic retina during proliferative vitreoretinopathy. Glia.

[26] Yoshimura T, Sonoda KH, Sugahara M, Mochizuki Y, Enaida H, Oshima Y, Ueno A, Hata Y, Yoshida H, Ishibashi T (2009). Comprehensive analysis of inflammatory immune mediators in vitreoretinal diseases. PLoS ONE.

[27] Wladis EJ, Falk NS, Iglesias BV, Beer PM, Gosselin EJ (2013). Analysis of the molecular biologic milieu of the vitreous in proliferative vitreoretinopathy. Retina.

[28] Pollreisz A, Sacu S, Eibenberger K, Funk M, Kivaranovic D, Zlabinger GJ, Georgopoulos M, Schmidt-Erfurth U (2015). Extent of Detached Retina and Lens Status Influence Intravitreal Protein Expression in Rhegmatogenous Retinal Detachment. Invest Ophthalmol Vis Sci.

[29] Kim B, Abdel-Rahman MH, Wang T, Pouly S, Mahmoud AM, Cebulla CM (2014). Retinal MMP-12, MMP-13, TIMP-1, and TIMP-2 expression in murine experimental retinal detachment. Invest Ophthalmol Vis Sci.

[30] Zajac E, Schweighofer B, Kupriyanova TA, Juncker-Jensen A, Minder P, Quigley JP, Deryugina EI (2013). Angiogenic capacity of M1-and M2-polarized macrophages is determined by the levels of TIMP-1 complexed with their secreted proMMP-9. Blood.

[31] Jiang K, Wright KL, Zhu P, Szego MJ, Bramall AN, Hauswirth WW, Li Q, Egan SE, McInnes RR (2014). STAT3 promotes survival of mutant photoreceptors in inherited photoreceptor degeneration models. Proc Natl Acad Sci U S A.

[32] Kyosseva SV (2016). Targeting MAPK Signaling in Age-Related Macular Degeneration. Ophthalmol Eye Dis.

[33] Dabritz J, Judd LM, Chalinor HV, Menheniott TR, Giraud AS (2016). Altered gp130 signalling ameliorates experimental colitis via myeloid cell-specific STAT3 activation and myeloid-derived suppressor cells. Sci Rep.

[34] Cao W, Wen R, Li F, Lavail MM, Steinberg RH (1997). Mechanical injury increases bFGF and CNTF mRNA expression in the mouse retina. Exp Eye Res.

[35] Samardzija M, Wenzel A, Aufenberg S, Thiersch M, Reme C, Grimm C (2006). Differential role of Jak-STAT signaling in retinal degenerations. FASEB J.

[36] Li R, Wen R, Banzon T, Maminishkis A, Miller SS (2011). CNTF mediates neurotrophic factor secretion and fluid absorption in human retinal pigment epithelium. PLoS ONE.

[37] Leibinger M, Muller A, Andreadaki A, Hauk TG, Kirsch M, Fischer D (2009). Neuroprotective and axon growth-promoting effects following inflammatory stimulation on mature retinal ganglion cells in mice depend on ciliary neurotrophic factor and leukemia inhibitory factor. J Neurosci.

[38] Kirsch M, Trautmann N, Ernst M, Hofmann HD (2010). Involvement of gp130-associated cytokine signaling in Muller cell activation following optic nerve lesion. Glia.

[39] Lin HW, Jain MR, Li H, Levison SW (2009). Ciliary neurotrophic factor (CNTF) plus soluble CNTF receptor alpha increases cyclooxygenase-2 expression, PGE2 release and interferon-gamma-induced CD40 in murine microglia. J Neuroinflammation.

[40] Miotke JA, MacLennan AJ, Meyer RL (2007). Immunohistochemical localization of CNTFRalpha in adult mouse retina and optic nerve following intraorbital nerve crush: evidence for the axonal loss of a trophic factor receptor after injury. J Comp Neurol.

[41] Bucher F, Walz JM, Buhler A, Aguilar E, Lange C, Diaz-Aguilar S, Martin G, Schlunck G, Agostini H, Friedlander M, Stahl A (2016). CNTF Attenuates Vasoproliferative Changes Through Upregulation of SOCS3 in a Mouse-Model of Oxygen-Induced Retinopathy. Invest Ophthalmol Vis Sci.

[42] Larsen JV, Kristensen AM, Pallesen LT, Bauer J, Vaegter CB, Nielsen MS, Madsen P, Petersen CM (2016). Cytokine-Like Factor 1, an Essential Facilitator of Cardiotrophin-Like Cytokine:Ciliary Neurotrophic Factor Receptor alpha Signaling and sorLA-Mediated Turnover. Mol Cell Biol.

[43] Wang D, Liu L, Yan J, Wu W, Zhu X, Wang Y (2015). Cardiotrophin-1 (CT-1) improves high fat diet-induced cognitive deficits in mice. Neurochem Res.

[44] Burton MD, Rytych JL, Freund GG, Johnson RW (2013). Central inhibition of interleukin-6 trans-signaling during peripheral infection reduced neuroinflammation and sickness in aged mice. Brain Behav Immun.

[45] Haroon F, Drogemuller K, Handel U, Brunn A, Reinhold D, Nishanth G, Mueller W, Trautwein C, Ernst M, Deckert M, Schluter D (2011). Gp130-Dependent Astrocytic Survival Is Critical for the Control of Autoimmune Central Nervous System Inflammation. J Immunol.

[46] Van Hove I, Lefevere E, De Groef L, Sergeys J, Salinas-Navarro M, Libert C, Vandenbroucke R, Moons L. MMP-3 Deficiency Alleviates Endotoxin-Induced Acute Inflammation in the Posterior Eye Segment. Int J Mol Sci. 2016;17. 10.3390/ijms17111825 PMC5133826 27809288

[47] Dvoriantchikova G, Barakat DJ, Hernandez E, Shestopalov VI, Ivanov D (2010). Liposome-delivered ATP effectively protects the retina against ischemia-reperfusion injury. Mol Vis.

[48] Rochet E, Brunet J, Sabou M, Marcellin L, Bourcier T, Candolfi E, Pfaff AW (2015). Interleukin-6-driven inflammatory response induces retinal pathology in a model of ocular toxoplasmosis reactivation. Infect Immun.

[49] Lee JJ, Wang PW, Yang IH, Huang HM, Chang CS, Wu CL, Chuang JH (2015). High-fat diet induces toll-like receptor 4-dependent macrophage/microglial cell activation and retinal impairment. Invest Ophthalmol Vis Sci.

[50] Matsumoto H, Kataoka K, Tsoka P, Connor KM, Miller JW, Vavvas DG (2014). Strain difference in photoreceptor cell death after retinal detachment in mice. Invest Ophthalmol Vis Sci.

[51] Kimura K, Orita T, Liu Y, Yang Y, Tokuda K, Kurakazu T, Noda T, Yanai R, Morishige N, Takeda A (2015). Attenuation of EMT in RPE cells and subretinal fibrosis by an RAR-gamma agonist. J Mol Med (Berl).

[52] Matsumoto H, Murakami Y, Kataoka K, Lin H, Connor KM, Miller JW, Zhou D, Avruch J, Vavvas DG (2014). Mammalian STE20-like kinase 2, not kinase 1, mediates photoreceptor cell death during retinal detachment. Cell Death Dis.

[53] Kunikata H, Yasuda M, Aizawa N, Tanaka Y, Abe T, Nakazawa T (2013). Intraocular concentrations of cytokines and chemokines in rhegmatogenous retinal detachment and the effect of intravitreal triamcinolone acetonide. Am J Ophthalmol.

[54] Symeonidis C, Androudi S, Tsaousis KT, Tsinopoulos I, Brazitikos P, Diza E, Dimitrakos SA (2012). Comparison of interleukin IL-6 levels in the subretinal fluid and the vitreous during rhegmatogenous retinal detachment. Cytokine.

[55] Chong DY, Boehlke CS, Zheng QD, Zhang L, Han Y, Zacks DN (2008). Interleukin-6 as a photoreceptor neuroprotectant in an experimental model of retinal detachment. Invest Ophthalmol Vis Sci.

[56] Curnow SJ, Scheel-Toellner D, Jenkinson W, Raza K, Durrani OM, Faint JM, Rauz S, Wloka K, Pilling D, Rose-John S (2004). Inhibition of T cell apoptosis in the aqueous humor of patients with uveitis by IL-6/soluble IL-6 receptor trans-signaling. J Immunol.

[57] Echevarria FD, Rickman AE, Sappington RM. Interleukin-6: A Constitutive Modulator of Glycoprotein 130, Neuroinflammatory and Cell Survival Signaling in Retina. J Clin Cell Immunol. 2016;7. 10.4172/2155-9899.1000439 PMC5061045 27747134

[58] Sims SM, Holmgren L, Cathcart HM, Sappington RM (2012). Spatial regulation of interleukin-6 signaling in response to neurodegenerative stressors in the retina. Am J Neurodegener Dis.

[59] Hsu MP, Frausto R, Rose-John S, Campbell IL (2015). Analysis of IL-6/gp130 Family Receptor Expression Reveals That in Contrast to Astroglia, Microglia Lack the Oncostatin M Receptor and Functional Responses to Oncostatin M. Glia.

[60] Rose-John S (2012). IL-6 trans-signaling via the soluble IL-6 receptor: importance for the pro-inflammatory activities of IL-6. Int J Biol Sci.

[61] Nagineni CN, Kommineni VK, William A, Hooks JJ, Detrick B (2010). IL-11 expression in retinal and corneal cells is regulated by interferon-gamma. Biochem Biophys Res Commun.

[62] Cho N, Nguyen DH, Satkunendrarajah K, Branch DR, Fehlings MG (2012). Evaluating the role of IL-11, a novel cytokine in the IL-6 family, in a mouse model of spinal cord injury. J Neuroinflammation.

[63] Lokau J, Agthe M, Garbers C (2016). Generation of Soluble Interleukin-11 and Interleukin-6 Receptors: A Crucial Function for Proteases during Inflammation. Mediators Inflamm.

[64] Lee YS, Amadi-Obi A, Yu CR, Egwuagu CE (2011). Retinal cells suppress intraocular inflammation (uveitis) through production of interleukin-27 and interleukin-10. Immunology.

[65] Senecal V, Deblois G, Beauseigle D, Schneider R, Brandenburg J, Newcombe J, Moore CS, Prat A, Antel J, Arbour N (2016). Production of IL-27 in multiple sclerosis lesions by astrocytes and myeloid cells: Modulation of local immune responses. Glia.

[66] Wang M, Ma W, Zhao L, Fariss RN, Wong WT (2011). Adaptive Muller cell responses to microglial activation mediate neuroprotection and coordinate inflammation in the retina. J Neuroinflammation.

[67] Chucair-Elliott AJ, Elliott MH, Wang J, Moiseyev GP, Ma JX, Politi LE, Rotstein NP, Akira S, Uematsu S, Ash JD (2012). Leukemia inhibitory factor coordinates the down-regulation of the visual cycle in the retina and retinal-pigmented epithelium. J Biol Chem.

[68] Lange C, Thiersch M, Samardzija M, Burgi S, Joly S, Grimm C (2010). LIF-dependent JAK3 activation is not essential for retinal degeneration. J Neurochem.

[69] Rattner A, Toulabi L, Williams J, Yu H, Nathans J (2008). The genomic response of the retinal pigment epithelium to light damage and retinal detachment. J Neurosci.

[70] Moidunny S, Matos M, Wesseling E, Banerjee S, Volsky DJ, Cunha RA, Agostinho P, Boddeke HW, Roy S (2016). Oncostatin M promotes excitotoxicity by inhibiting glutamate uptake in astrocytes: implications in HIV-associated neurotoxicity. J Neuroinflammation.

[71] Delyfer MN, Raffelsberger W, Mercier D, Korobelnik JF, Gaudric A, Charteris DG, Tadayoni R, Metge F, Caputo G, Barale PO, et al. Transcriptomic Analysis of Human Retinal Detachment Reveals Both Inflammatory Response and Photoreceptor Death. Plos One. 2011;6. 10.1371/journal.pone.0028791 PMC3235162 22174898

[72] Hams E, Colmont CS, Dioszeghy V, Hammond VJ, Fielding CA, Williams AS, Tanaka M, Miyajima A, Taylor PR, Topley N, Jones SA (2008). Oncostatin M receptor-beta signaling limits monocytic cell recruitment in acute inflammation. J Immunol.

[73] Drechsler J, Grotzinger J, Hermanns HM. Characterization of the Rat Oncostatin M Receptor Complex Which Resembles the Human, but Differs from the Murine Cytokine Receptor. Plos One. 2012;7. 10.1371/journal.pone.0043155 PMC3425591 22937020

