# Supplementary material for: Rapid monocyte infiltration following retinal detachment is dependent on non-canonical IL6 signaling through gp130
Source: J Neuroinflammation. 2017 Jun 23;14:121. doi: 10.1186/s12974-017-0886-6 (PMC5481880; doi:10.1186/s12974-017-0886-6)
Supplement: Supplementary file 1 — Table S1. Antibodies used in the current study. (PDF 237 kb) [file 12974_2017_886_MOESM1_ESM.pdf]

Supplementary Table 1. Antibodies used in the current study

Antibody	Company	Name and Catalogue Numbers	Source	Concentration (μg/μl)	Epitope Mapping	Applications in current study	Results
Anti-IL6Rα	**Santa Cruz**	M-20, sc-660	rabbit polyclonal IgG	0.2	C-terminus of mouse IL6Rα	IF, WB	IL6Rα is undetactable in retina
		D-8, sc-374259	mouse monoclonal IgG1	0.2	amino acids 433-460 at the C-terminus of mouse IL6Rα		
	**R&D Systems**	AF1830	goat polyclonal IgG	0.362-1, original concentration of different lot numbers	amino acids 20-357 of mouse myeloma cell line NS0-derived recombinant IL6Rα	IF, WB, Neutralization	IL6Rα is undetactable in retina; failed to prevent CD11b+ monocytes after RD by intravitreal injection
	**LifeSpan BioSciences**	extracellular domain, LS-C70920	rat monoclonal IgG1	0.5	amino acids 20-357 of mouse myeloma cell line NS0-derived recombinant IL6Rα	WB	IL6Rα is undetactable in retina, but positive in lysates of liver
Anti-gp130	**R&D Systems**	MAB4682	rat monoclonal IgG1	0.362-1, original concentration of different lot numbers	amino acids 23-617 of mouse myeloma cell line NS0-derived recombinant gp130	Neutralization	Anti-gp130 treatment decreased retinal CCL2 level, number of CD11b+ monocytes, as well as inhibited Erk1/2 expression and stimulated STAT3 upregulation in aqueous humor/vitreous samples
	**EMD Millipore**	06-291	rabbit polyclonal IgG	1	amino acids 713-918	WB	gp130 protein is detected in retina and auqeous humor/ vitreous with double bands
	**Cell signaling Technology**	3732S	rabbit polyclonal	100µl	endogenous levels of total GP130 protein independent of phosphorylation	WB	
	**Santa Cruz**	M-20, sc-656	rabbit polyclonal IgG	0.2	C-terminus of mouse gp130 origin	WB, IF	double bands observed on western blot; in immunofluorescence staining, gp130 expression was initially confined to outer nuclear layer of normal retina, and extended into the inner nuclear layer and monocytes in vitreous humor after RD
**IgG1 isotype control**	**R&D Systems**	MAB002	monoclonal mouse IgG1 isotype control	1	N/A	Flow cytometry, IF	IgG1 isotype control showed more CD11b+ monocytes recruited into retina compared with gp130 blocking one day after RD.

IF: Immunofluorescence RD: retinal detachment WB: western blot
