# Supplementary material for: Rapid monocyte infiltration following retinal detachment is dependent on non-canonical IL6 signaling through gp130
Source: J Neuroinflammation. 2017 Jun 23;14:121. doi: 10.1186/s12974-017-0886-6 (PMC5481880; doi:10.1186/s12974-017-0886-6)
Supplement: Supplementary file 2 — Table S2. Ocular Expression of the gp130 Receptor Cytokine Family in Normal and Detached Retina of Mouse and Human. (PDF 219 kb) [file 12974_2017_886_MOESM2_ESM.pdf]

Supplementary Table 2. Ocular Expression of the gp130 Receptor Cytokine Family in Normal and Detached Retina of Mouse and Human

Cytokines & Receptors	Normal Mouse/Human Eye			Mouse/Human RD/PVR			Roles in Neuroinflammation	Roles in gp130 Signaling Pathway
	mRNA	IHC	Western Blot/ ELISA/ Cytokine arrays	mRNA	IHC	Western Blot/ ELISA/ Cytokine arrays		
**CNTF**	mouse retina[34, 35]; relatively low in hfRPE[36]	weakly expressed in mouse GFAP positive-astrocytes/ Müller cells[37]	mouse retina[37]	N/A	N/A	N/A	CNTF was not detected in retinal microglia or macrophages. lens injury-induced macrophages infiltration, activation of microglia and astrocytes/Müller cells were not reduced in CNTF KO mice[37]	CNTF knock-out didn't affect retinal LIF expression via western blot after ONC[37]. CNTF KO doesn't affect mRNA expressions of CNTFRα, LIF, LIFRβ, gp130, nor interfere STAT3 phosphorylation and SOCS3 activation [38]. CNTF did not activate phosphorylation of STAT-3, ERK, nor p38 MAP kinase in brain microglia [39]
**CNTFR**	no expression in most mouse retinal cells [18]; mouse retina[34]	intensely expressed in mouse GCL and NFL[40]; detected in OS and GCL[41]	undetectable in hfRPE[36]	N/A	N/A	N/A	CNTF weakly stimulated microglia, whereas soluble CNTFR alpha strongly did [39]	CLC:CLF-1:CNTFRα complex on cell membranes signals through gp130/LIFRβ[42]
**Cardiotrophin-1**	mouse retina[35]; hfRPE[36]	N/A	N/A	N/A	N/A	N/A	alleviate high-fat diet induced cognitive impairment[43]	increase STAT3 phosphorylation in hfRPE[36]
**gp130**	highly expressed in hfRPE[36]	mouse ONL*	hfRPE[36], mouse retina*	N/A	increased in mouse INL and vitreous CD11b positive-monocytes*	significantly increase in mouse vitreous, not in retina after RD*	soluble gp130 exerts an anti-inflammatory effect by inhibiting the pro-inflammatory arm of IL-6 signaling in the aged brain [44]	prevention of astrocyte apoptosis, restriction of demyelination, and T cell infiltration were dependent on the gp130-Src homology region 2 domain-containing phosphatase 2/Ras/ERK, but not on the gp130-STAT1/3 pathway[45]
**IL6**	no expression in most mouse retinal cells [18]; undetectable in mouse retina and RPE [46]	Low-undetectable in mouse retina[47-49]	undetectable in mouse retina* and very low in human MIO-M1 by multiple arrays[25]; <5pg/ml in mouse retina [50, 51] and <300pg/ml in mouse RPE cells [52] by ELISA	increased in the vitreous samples of PVR patients[10]	N/A	12 folds increase in the aqueous humor of RD patients[53], 5-15 folds and 23 folds increase in the vitreous of RD and PVR patients[27,54], 2.5 folds increase in subretinal fluid of PVR patients compared with RD patients[8] by ELISA; no increase in mouse retina after RD*, but increased in human PVR retina [25] by multiple arrays	IL6 serve as photoreceptor neuroprotectant [55]; combinations of IL-6 and soluble IL-6R effectively inhibited apoptosis of T cell [56]. microglia are important regulators of the IL-6 trans-signaling response in the aged brain [44]	IL6 KO failed to affect mRNA of gp130, but reduced gp130 protein by 30% in mouse retina[57]
**IL6Rα**	no expression in most mouse retinal cells [18]	weak localization to RGC soma and vasculature [58]	undetectable in mouse retina by multiple arrays*; very low in mouse retina measured by western blot[58]	N/A	N/A	membrane-bound IL6Rα was undetectable in mouse retina and vitreous after RD by western blot*	IL-6R mRNA and protein was present in mouse brain microglia[59]	IL-6 trans-signaling is pro-inflammatory whereas classic IL-6 signaling via the membrane bound IL-6R is needed for regeneration or anti-inflammatory activities[60]
**IL11**	no expression in most mouse retinal cells [18]; weak in human RPE[61]	N/A	N/A	N/A	N/A	no increase in human PVR retina[25] by multiple arrays; no change in subretinal fluid between RD and PVR patients by ELISA[8]	IL-11 signaling may not play as significant a role as other gp130 cytokines in mouse model of spinal cord injury. [62]	IL-11 can signal via trans-signaling mechanism[63]
**IL11Rα**	high expression in most mouse retinal cells [18]	N/A	N/A	N/A	N/A	N/A	brain microglia has lower IL-11R mRNA[59].	
**IL27**	mouse retina[64]	F4/80 positive-microglia in mouse INL and GCL[64]	very low in human MIO-M1 by multiple arrays[25]	N/A	N/A	no increase in mouse retina after RD* and human PVR retina[25] by multiple arrays	inhibited Th17 development and expansion in experimental autoimmune uveitis[64]; local IL-27 can modulate infiltrating immune cells[65]	Exogenous IL-27 increased STAT1 phosphorylation, and up-regulated mRNAa of SOCS1[64]
**IL27Rα/WSX-1**	no expression in most mouse retinal cells [18]; weak in mouse[64]	mouse OS[64]	N/A	N/A	N/A	N/A		
**LIF**	no expression in most mouse retinal cells [18]; weak in primary mouse Müller cells[66]; mouse retina[35]	weakly expressed in GFAP positive-mouse astrocytes/ Müller cells[37]	weak in mouse retina[37]; <25pg/ml in primary mouse Müller cells by ELISA[66]	N/A	N/A	no increase in human PVR retina[25] by multiple arrays; no change in vitreous of RD and PVR patients by ELISA[67]	lens injury-induced macrophages infiltration and activation of microglia and astrocytes/Müller cells were not reduced in CNTF/LIF double KO mice[37]. brain microglia has lower LIFR mRNA[59]	pSTAT3 increased after exogenous LIF treatment[68]. Light induced upregulation of Jak3 was absent in LIF KO mice[69]
**LIFR**	no expression in most mouse retinal cells [18]; relatively low in hfRPE[36]	mainly on the apical membrane of hfRPE[36]	positive in hfRPE[36] by western blot	N/A	N/A	N/A		
**Oncostatin M (OSM)**	no expression in most mouse retinal cells [18]; relatively low in hfRPE[36]	undetectable in mouse retina, weakly expressed in RPE[70]	N/A	N/A	N/A	N/A	activation of JAK/STAT3 signaling by OSM results in neuronal excitotoxicity[71]	induces STAT3 and ERK1/2 phosphorylation in hfRPE[36]
**OSMR**	no expression in most mouse retinal cells [18]; mouse retina[70]	undetectable in mouse retina, weakly expressed in RPE[70]	undetectable in mouse retina[70]; positive in hfRPE[36] by western blot	increased in human RD[72]	localized increase in mouse INL and RPE[70]	N/A	increased in the apical microvilli of the RPE and OS region after light demage[70]; present in brain astrocytes but was undetectable in brain microglia[59]; OSMR-KO mice show increased monocyte trafficking during acute inflammation[73]	activate the Jak/STAT, the MAP kinase as well as the PI3K/Akt pathways[74]

CLC: cardiotrophin-like cytokine
CLF-1: cytokine-like factor-1
GCL: ganglion cell layer
hfRPE: human fetal RPE
INL: inner nuclear layer
KO: knock out
MIO-M1: human Müller cell line
N/A: no answer
NFL: nerve fiber layer
ONC: optic nerve crush
ONL: outer nuclear layer
OS: outer segments of photoreceptor

*shown in results of the current study
